# Interventions for Body Composition and Upper and Lower Extremity Muscle Strength in Older Adults in Rural Taiwan: A Horizontal Case Study

**DOI:** 10.3390/ijerph19137869

**Published:** 2022-06-27

**Authors:** Chun-An Chen, Ming-Chi Lai, Hsuan Huang, Cheng-En Wu

**Affiliations:** 1 Ph.D. Program of Technology Management, Chung Hua University, Hsinchu 30012, Taiwan; cachen@chu.edu.tw (C.-A.C.); d10903006@chu.edu.tw (C.-E.W.); 2 Department of Occupational Therapy, National Cheng Kung University, Tainan 701401, Taiwan; i74060011@ncku.edu.tw

**Keywords:** rural, older adults, body composition, physical activity program, high-protein supplementation

## Abstract

The purpose of this study was to understand the effects of a physical activity program and high-protein supplementation on body composition and upper and lower extremity muscle strength in male older adults in rural areas. In this study, 60 healthy male older adults (mean age 77.5 ± 4.6 years) from rural areas were recruited and randomly assigned to experimental group A (intervention of the physical activity program and high-protein supplementation), experimental group B (daily routine, with only intervention of high-protein supplementation), or control group C (daily routine). Experimental group A (EGa) carried out a physical activity plan three times a week, with an exercise intensity and calorie consumption of 250 kcal (5METs × ⅔hr × 75) for 3 months and drank a high-protein supplement (1.3 g/kg BW/day) after each exercise; experimental group B (EGb) followed only the intervention of high-protein supplementation. All the participants underwent pre- and post-tests for body composition, waist–hip circumference (WC, HC), handgrip strength (HS), 30 s dominant arm curl, 30 s sit to stand, and 2 min step tests. The results of the study showed that EGa significantly decreased body mass index (BMI), body fat mass (BFM), body fat percentage (BFP), WC, HC, and waist-to-hip ratio (WHR) and increased basal metabolic rate and muscle mass. Although both EGa and EGb used high-protein supplementation, EGa’s added three-month intervention of a physical activity program made it easier for that group to increase muscle mass and muscle strength. The WHR decreased from 1.015 to 0.931, representing a decrease of 8.28%, and an obvious weight loss effect was achieved. Thus, we concluded that the best way to maintain muscle strength in older adults is through physical activity with resistance and protein supplementation, which can reduce muscle loss in older adults.

## 1. Introduction

Taiwan will become a super-aged society by 2025, and the population over 65 will account for about 20% of the total. In other words, one out of every four or five people will be an older adult [1]. It is expected that 26% of Taiwan’s total population will be older adults by 2060 [2]. Geographically, there are currently 23 townships in Taiwan, and more than 20% of the local residents are over the age of 65 [3]. Most of Taiwan’s townships are located in remote areas, and young people move to urban areas for jobs and schools. These remote townships with high proportions of aging residents have formed many long-term care institutions. Nursing care for the older adults in remote townships faces the challenges of a lack of medical human resources and the fact that with more Taiwanese women participating in the labor force, women have less time to take care of their families [1,4]. Long-term care centers in remote areas are being formed, and the lack of nursing staff and medical rehabilitation personnel has become a major challenge to the care and health of the older adults living in long-term care centers.

Rural Taiwan contains natural landscapes, farmland, sloping land, forests, rivers, villages, small towns, and industrial centers. The people living in it are mainly farmers engaged in agricultural activities. In a broad sense, it also includes residents who engage in non-agricultural activities such as fishery, forestry, and animal husbandry [5]. The minimum population size of Taiwan’s rural settlements is 4000 people, and the maximum can be as high as tens of thousands. The distance between each village is 2 to 5 km. With the expansion of cities and the improvement of transportation, the distinction between “urban” and “rural” has become more blurred over time. Many young people or children in rural areas work or study in neighboring cities and towns. The number of full-time farmers has been decreasing daily. Most of the residents living in rural areas are older adults [6]. According to the World Health Organization’s publication *Global Age-friendly Cities: Guide in 2007*, Taiwan chose Chiayi as its pilot age-friendly city for older adults in 2010. By 2013, 22 counties and cities in Taiwan had implemented age-friendly programs [7], and today, the business operations of the long-term care facilities established in Chiayi have reached maturity. For this study, the Zhongpu Township Cibao Elderly Care Center, which has been operating for 20 years, was selected. Longxing Village, Zhongpu Township, Chiayi County is a rural area in Taiwan. According to statistics from the Shuishang Household Registration Office for Chiayi County [8], there are 8713 older adults over the age of 65 in Zhongpu Township (as of April 2022), accounting for 19.98% of the total population of 43,602 people. Longxing Village in Zhongpu Township has a total of 405 older adults over the age of 65, accounting for 19.82% of the total population of Longxing Village (2043 older adults). It can be seen from the above that the ratio of older adults in rural Taiwan is approaching that of a super-aged society.

At present, the proportion of older adults in rural Taiwan is higher than that in urban areas [9], and the older adults in rural areas have insufficient knowledge of health care and are not aware of the extended physiological symptoms of aging. Many studies have shown that aging is often accompanied by insufficient muscle mass [10,11,12], and studies have found that after the age of 30, muscle mass begins to become deficient, with an average decline of 3–5% per year. After the age of 50, the levels of hormones begin to decline and muscle mass declines more rapidly, increasing by a factor of 2 [13,14]. Kalyani et al. reported that the most obvious problems caused by a lack of muscle mass are the lack of muscular endurance and physical strength, leading to older adults becoming prone to falls and thus worsening their risk of fracture or death [15]. This may also lead to cardiovascular disease fat accumulation in the body, resulting in obesity or other chronic diseases [16].

Many studies have shown that middle-aged and older adults should monitor their nutritional intake and perform regular exercise to maintain muscle and avoid sarcopenia problems [17,18,19,20]. In terms of nutritional intake, Paddon-Jones and Leidy suggest that consuming adequate dietary protein intake is key to maintaining muscle mass and quality of life in older adults [21]. Nowson and O’Connell recommend that the daily protein intake of older adults be 1.2 g per kilogram per day (1.2 g/kg/day) [22]. For example, an adult weighing 70 kg should consume at least 84 g of protein per day to maintain muscle mass. Protein is an essential nutrient for muscles, and adequate protein intake can help prevent sarcopenia [23]. In terms of exercise, muscle mass in older adults declines at a rate of 3% per year after the age of 80. Exercise causes the muscles to contract, stimulates brain activity, and prevents dementia [24]. According to Campbell et al., the positive effects of exercise on quality of life (QoL) and the activities of daily living (ADL) in frail older adults have been well documented [25]. The WHO recommends that older adults exercise as much as their abilities and conditions permit and perform aerobic exercise, strength training, and balance exercises to reduce their risk of falls [26]. The ACSM has similar recommendations for at least 150 min of moderate-intensity physical activity per week for 65-year-olds [27]. Taylor found that exercise had a positive effect on mobility and physical performance in healthy older adults and that higher-intensity exercise was more effective than low-intensity exercise for improving physical function [28]. Chou et al. also demonstrated that strength training interventions were important for improving physical function [29].

Based on the above literature, it can be surmised that older adults living in rural areas have insufficient knowledge of health care and regard manual labor as exercise. There is still insufficient research on the use of physical activity programs and high-protein supplements in older adults, and the use of older adults in rural areas as research subjects is even rarer. This is what makes this study unique. Therefore, the purpose of this study was to focus on improving the body composition and upper and lower limb strength of older adults in rural areas through an intervention consisting of a physical activity program and high-protein supplementation. Our research hypotheses were as follows. Hypothesis one (H1): The physical activity program and high-protein supplementation will improve participants’ body compositions and waist–hip circumferences. H2: The physical activity program with high-protein supplementation will improve participants’ upper and lower extremity muscle strength. H3: The physical activity program and high-protein supplementation will be effective for achieving weight loss and muscle gain.

## 2. Materials and Methods

### 2.1. Participants

In Taiwan, Chiayi was chosen as the pilot age-friendly city for older adults in 2010. Therefore, in this study we used older adults from the Cibao Elderly Care Center in Longxing Village, Zhongpu Township, Chiayi County, as the research participants. Since the study participants were affiliated with the Cibao Elderly Care Center in Longxing Village, Zhongpu Township, Chiayi County, 60 healthy older adults were recruited as volunteers to participate in this research project (the term “healthy older adults” refers to those who have good physical functions, can take care of themselves completely in their daily lives, retain their original mobility and social skills, and have the ability to move freely and live in self-determination [30]; the term “mobile healthy older adults” refers to those who can walk or run independently in horizontal and vertical space without relying on assistive devices according to the standard of daily living). For all participants, we gathered basic information consisting of age, height, and weight and filled out a health certificate (exclusion criteria: (1) medical problems or physical limitations preventing physical activity; (2) those in wheelchairs or undergoing dialysis; (3) those suffering from serious injury or illness; (4) those who have physical or mental conditions that prevent them from participating in nutrition or exercise). All participants signed an informed consent form according to their own wishes (contents included: 3 months in addition to a standardized lunch, followed by a complete daily diet. In addition to experimental groups A and B, the control group was prohibited from taking supplements that could increase their muscle mass). The participants were randomly divided into three groups. The first group was experimental group A (average age 77.2 ± 5.3 years), the second group was experimental group B (average age 78.5 ± 6.7 years), and the third group was control group C (average age 76.9 ± 6.1 years). The three groups completed the F test, age (F = 0.94, *p* > 0.05), height (F = 1.13, *p* > 0.05), and weight (F = 1.07, *p* > 0.05). There were no significant differences in the F values for the three groups above, indicating that the results obtained from the random allocation of participants were homogeneous, as shown in Table 1. All three groups of participants were non-smokers, had no orthopedic problems or heart disease, and were able to go about their daily lives without assistance. All procedures were performed in accordance with institutional and/or national research council ethical standards and the 1964 Declaration of Helsinki and its subsequent revisions or similar ethical standards. Study procedures and protocols were approved by the Institutional Review board (110-96). All the questions were addressed, and participants signed informed consent prior to the assessment.

### 2.2. Research Material

The participants were randomly divided into three groups. The first group was experimental group A (intervention with the physical activity program and high-protein supplementation), the second group was experimental group B (daily routine with only intervention with high-protein supplementation), and the third group was control group C (daily routine). The following is a description of the activities performed by each group for three months.

#### 2.2.1. Intervention of Physical Activity Program

The exercise intervention described in this study consisted of a physical activity program based on the Physical Activity Guidelines for Americans (PAGA) [31]. The intensity of the physical activity program is displayed in metabolic equivalents (METs), where the so-called MET is defined as the consumption of 3.5 mL of oxygen per kilogram of body weight per minute, which is roughly equivalent to the consumption of oxygen per minute when a person sits in a quiet state without performing any activity. An activity level of 5 METs means that the oxygen consumption during exercise is 5 times that of the resting state [32], meaning that the intensity and calorie consumption of each physical activity in experimental group A (EGa) was 250 kcal (5METs × ⅔hr × 75) and the intensity was moderate. This physical activity program was personally run by the researchers, and the participants engaged in physical activity 3 times per week. The program ran for 3 months, and all participants in EGa attended 95% or more of the classes. Each time the physical activity program was executed, 6 movements were used as a cycle, and each movement was performed for 1 min (about 10–20 times), for a total of 3 cycles. The program included step-ups, chair squats, pistol squats, standing lunges, walking in place with high legs, 10-pound dumbbell arm curls, 10-pound dumbbell flyers, and 10-pound dumbbell shoulder raises. If the participants were unable to complete their own exercise, they could be assisted by holding the back of a chair with both their hands (standing lunge: two chair backs are placed on the side of the body, and the chair is supported by the participants’ back during operation). The details of the physical activity program are shown in Table 2.

#### 2.2.2. Intervention of High-Protein Supplementation

In this study, experimental groups A (EGa) and B (EGb) were given high-protein supplementation three times a week. EGa drank high-protein supplementation 10 minutes after completing the physical activity program (1.3 g/kg BW/day, 68 kg × 1.3 g = 88 g protein per day) [33,34], while EGb performed daily routines (such as reading newspapers and magazines, watching TV, walking, chatting, playing chess) and drank the high-protein supplementation 3 times a week. The intervention lasted for 3 months. Control group C (CGc) performed daily routines without any intervention. Three groups of daily lunches were provided by Cibao Elderly Care Center for a total of three months.

### 2.3. Detection Method

The 60 participants in this study had to pass pre-test and post-test analyses. The test contents were divided into body composition tests and waist and hip circumference measurements. In addition, for the measurement of upper and lower limb muscle strength, we referred to the senior fitness test (SFT) developed by Rikli and Jones [35]. The items tested in this study were handgrip strength, 30 s dominant arm curl, 30 s sit to stand, and 2 min step, as shown in Table 3.

### 2.4. Statistical Analysis

Descriptive statistics were performed. Shapiro–Wilk tests were performed on all dependent variables for the three sets of pre- and post-test data to check the assumption of normality. The results showed that all data support the assumption of normal distribution. The results are expressed as means and standard deviation (±) values. Comparisons between the three groups were performed using one-way ANOVA and the LSD post hoc test (applicable to many-to-one comparisons; when the number of tests is small and the significance level is small, we can use the overall significance level of *p* < 0.05). The significance level was set at *p* < 0.05. Cohen’s d was calculated to determine the effect size of the t-test and the adequacy of the sample size [38]. Statistical analysis was performed using the SPSS 20.0 software (IBM^®^, Armonk, NY, USA).

## 3. Results

### 3.1. Body Composition Measurement

**Figure 1:**
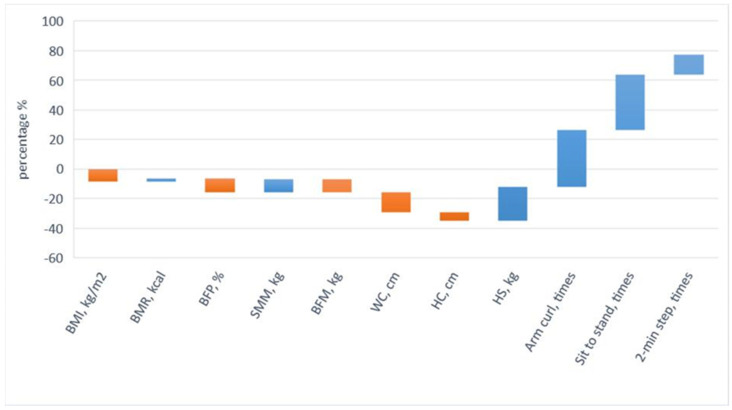
The EGa intervention results.

The pre-test and post-test results of the body composition factors of the EGa, EGb, and CGc are shown in Table 4 and Table 5. The results showed that the body composition of EGa was significantly different between the pre-test and post-test results (*p* < 0.05). On the contrary, the body composition of EGb and CGc were not significantly different between the pre-test and the post-test (*p* < 0.05). In the body composition experiment results for EGa, BMI decreased by 8.62% (t = 5.71, *p* < 0.05), BMR increased by 2.31% (t = 5.25, *p* < 0.05), BFP decreased by 9.30% (t = 6.37, *p* < 0.05), SMM showed an 8.90% improvement (t = 7.56, *p* < 0.05), and there was a 9.24% reduction in BFM (t = 5.45, *p* < 0.05). In addition, the post-test comparison results of the three groups were all significantly different, and then LSD post-test was performed, as shown in Table 6. BMI (F = 3.88, *p* < 0.05), BMR (F = 6.77, *p* < 0.05), BFP (F = 5.48, *p* < 0.05), SMM (F = 4.15, *p* < 0.05), and BFM (F = 3.68, *p* < 0.05). LSD of post hoc test, BMI (EGa > EGb = CGc), BMR (EGa > EGb = CGc), BFP (EGa > EGb = CGc), SMM (EGa > EGb = CGc), and BFM (EGa > EGb = CGc). To summarize the above, after a three-month intervention of a physical activity program and high-protein supplementation, EGa changed their overall body composition; significantly decreased their BMI, BFP, and BFM; and significantly increased their BMR and SMM. After three months of high-protein supplementation, EGb’s overall body composition improved slightly but not significantly. CGc’s body composition did not change after three months. The results validated H1: The physical activity program and high-protein supplementation improved participants’ body compositions and waist–hip circumferences, as shown in Figure 1.

### 3.2. Waist–Hip Circumference Measurement

The pre-test and post-test results obtained for the waist–hip circumferences of EGa, EGb, and CGc are shown in Table 4 and Table 5. The results showed that the waist–hip circumference of EGa was significantly different from the pre-test to the post-test (*p* < 0.05). On the contrary, there were no significant differences between EGb and CGc in the pre-test and post-test results (*p* < 0.05). The waist–hip circumference test results of EGa showed that the waist circumference decreased by 13.54% (t = 4.39, *p* < 0.05) and the hip circumference decreased by 5.70% (t = 5.38, *p* < 0.05). In addition, the post-test comparison results of the three groups were significantly different, followed by the LSD of the post hoc test, as shown in Table 6: waist circumference (F = 6.03, *p* < 0.05, EGa > EGb = CGc), hip circumference (F = 5.44, *p* < 0.05, EGa > EGb = CGc). In conclusion, EGa significantly reduced waist–hip circumference after the three-month intervention of a physical activity program and high-protein supplementation. There was no significant change in waist–hip circumferences for EGb and CGc after three months. The results validated H1: The physical activity program and high-protein supplementation improved the participants’ body compositions and waist–hip circumferences, as shown in Figure 1.

### 3.3. Muscle Strength Measurement of Upper and Lower Limbs

The pre-test and post-test results obtained for the upper and lower limb muscle strength of EGa, EGb, and CGc are shown in Table 4 and Table 5. The results show that the handgrip strength of EGa increased by 22.87% (t = 4.57, *p* < 0.05), the number of 30 s dominant arm curls increased by 38.48% (t = 8.82, *p* < 0.05), the number of 30 s sit to stand increased by 37.47% (t = 13.75, *p* < 0.05), and the number of 2 min steps increased by 13.47% (t = 11.43, *p* < 0.05). EGa increased handgrip strength and the number of 30 s dominant arm curls, showing that EGa increased their upper limb muscle strength. Both 30 s sit to stand and 2 min step values increased, showing increased EGa lower extremity muscle strength. The handgrip strength of EGb and the 2 min step of lower limbs were not significantly different between the pre-test and post-test; only the upper limb 30 s dominant arm curl (t = 3.12, *p* < 0.05) and the lower limb 30 s sit to stand (t = 3.07, *p* < 0.05) were significantly different between the pre-test and post-test. Conversely, there was no significant difference between the pre-test and post-test for the upper and lower extremity muscle strength of CGc.

In addition, the post-test comparison results of the three groups were all significantly different, and then the LSD of the post hoc test was performed, as shown in Table 6, with significant findings for handgrip strength (F = 3.85, *p* < 0.05), 30 s dominant arm curl (F = 5.26, *p* < 0.05), 30 s sit to stand (F = 5.71, *p* < 0.05), and 2 min step (F = 13.75, *p* < 0.05). LSD of post hoc test, handgrip strength (EGa > EGb = CGc), 30 s dominant arm curl (EGa > EGb > CGc), 30 s sit to stand (EGa > EGb > CGc), and 2 min step (EGa > EGb > CGc) EGa > EGb = CGc). In conclusion, after a three-month intervention of a physical activity program and high-protein supplementation, EGa significantly increased their muscle strength in their upper and lower limbs. After three months of high-protein supplementation in EGb, there was a slight improvement in the upper and lower extremity muscle strength. After three months of CGc, there was no significant change in the upper and lower extremity muscle strength. The results confirmed H2: The physical activity program with high-protein supplementation improved participants’ upper and lower extremity muscle strength, as shown in Figure 1.

### 3.4. Obesity Assessment

The pre-test and post-test results obtained for the waist–hip ratios (WHR) in EGa, EGb, and CGc are shown in Table 7. The results showed that only among EGa was there a significant effect on WHR (t = 3.714, *p* < 0.05), specifically a decrease of 8.28%. After the three-month intervention with the physical activity program and high-protein supplementation, the WHR of EGa decreased from 1.015 to 0.931 (World Health Organization standard for abdomen obesity for men: WHR ≥ 0.9). Table 4 shows that the BMI of EGa decreased from 27.48 kg/m^2^ to 25.11 kg/m^2^ (t = 5.71, *p* < 0.05), the BFP decreased from 27.97% to 25.37% (t = 6.37, *p* < 0.05), and the BFM decreased from 20.68 kg to 18.77 kg (t = 5.45, *p* < 0.05), indicating that EGa achieved weight loss. BMR increased from 1517 kcal to 1552 kcal (t = 5.25, *p* < 0.05), and SMM increased from 27.41 kg to 29.85 kg (t = 7.56, *p* < 0.05), showing that EGa increased muscle and metabolic capacity.

In terms of the WHR, that of EGb (2.61%) decreased more than that of CGc (0.59%), indicating that the intervention of the three-month high-protein supplementation produced a slight weight loss effect. The WHR post-test results of the three groups (F = 5.651, *p* < 0.05) showed significant differences, and in the LSD of the post hoc test, EGa > EGb > CGc; this finding indicated that the simultaneous use of physical activity plans and high-protein supplementation can promote weight loss and increase muscle. These results confirm H3: A physical activity program with high-protein supplementation was effective for weight loss and muscle gain.

## 4. Discussion

The participants in this study were all older adults from rural areas. Due to changes in their living environment and reductions in their number of family members [5], in addition to chatting with the older adults in the village, most of the older adults in rural areas said that they tended to sit at home for long periods, watch TV, and generally live monotonous daily lives [6]. This was consistent with the research of many scholars [1,39,40]. The lack of awareness of the benefits of exercise among older adults in rural Taiwan, resulting in increased physical frailty or obesity, was consistent with the findings of many scholars, and is believed to be related to a lack of physical exercise in this area [25,29,41,42]. The CGc in this study (WHR = 1.010~1.004, BMI = 27.22~27.41) was representative of older adults in rural Taiwan, and most of them had abdominal obesity, which was consistent with the research results of foreign scholars [43,44,45].

In this study, EGa after a 3-month physical activity program and high-protein supplementation showed enhancements in their upper and lower extremity muscle strength, consistent with our hypothesis regarding improved body composition. EGa participated in an intense physical activity program with calorie consumption of 250 kcal (5METs × ⅔hr × 75) with moderate-intensity exercise (3.0–5.9 METs) [32]. This type of activity can lead to slight tiredness, slightly faster breath and heartbeat than usual, and light sweating, and it meets the standard for healthy physical activity in older adults [46]. BMI, BFM, BFP, WC, HC, and WHR decreased significantly in EGa. Porter Starr et al. provide clinical evidence that exercise interventions can improve physical function in obese older adults [47]. The experimental results for EGa for BMR and SMM showed significantly increased basal metabolism and muscle mass. This result was in line with research conducted by Batsis et al. showing that the physical stimulation provided by exercise significantly improves muscle strength and physical function [43]. EGb and CGc only slightly sweated during their daily physical activities, but there were no significant changes in their body composition or in their upper and lower extremity muscle mass at three months. Some scholars have pointed out that because the human body is adapted to most daily activities in life, when the muscles are not stimulated by enough stress, it is difficult for them to increase in size [48,49].

Another important intervention in this study was the use of high-protein supplementation in EGa and EGb. The test results for EGb showed only slight changes in upper limb muscle strength (dominant arm curl, t = 3.12, *p* < 0.05) and lower limb muscle strength (30 s sit to stand, t = 3.07, *p* < 0.05). Numerous studies have confirmed the positive effects of high-protein supplementation on muscle and physical function in older adults. Houston et al. demonstrated that dietary protein intake was positively associated with maintaining lean body mass [50]. Tang et al. also demonstrated that the consumption of a high-protein diet helps to maintain lean body mass during weight loss [51]. Kim et al. confirmed that high-protein supplementation is beneficial for improving muscle mass in older adults [52]. Since EGb only received an intervention consisting of high-protein supplementation, they were only able to maintain or increase their muscle slightly (SMM, pre-test = 26.84 kg, post-test = 27.35 kg, improvement = 1.90%). EGa also used high-protein supplementation and achieved significant results (SMM, pre-test = 27.41 kg, post-test = 29.85 kg, improvement = 8.90%). Compared with EGb, EGa’s three months of a physical activity program was more likely to increase muscle mass and muscle strength. Chalé et al. demonstrated that high-protein supplementation in combination with exercise tends to increase lean body mass, thigh muscle cross-sectional area, and muscle strength [53]. Cermak et al. found that high-protein supplementation combined with resistance training enhanced strength and muscle mass in older adults [54]. Theodorakopoulos et al. determined that high-protein supplementation and exercise interventions are effective at improving body composition and muscle mass strength in older adults [55]. Liao et al. also demonstrated that high-protein supplementation and resistance training can effectively improve muscle strength and physical activity in older frail adults [56]. In addition, EGa and EGb consumed high-protein supplementation at the same time at around 10:00 a.m., consistent with the study by Candow and Chilibeck. The best time of day to consume protein is in the morning due to its closeness to bedtime, the generally lower activity levels after dinner, and the less efficient muscle synthesis occurring after protein intake [57]. Weinheimer et al. confirmed that taking protein supplements immediately after resistance exercise in middle-aged and elderly people can effectively increase SMM and promote metabolism [58].

The intervention of the physical activity program and high-protein supplementation utilized in this study effectively improved the upper and lower limb strength and optimized the body composition of older adults. The results of this study showed that after the intervention of the physical activity program and high-protein supplementation, the waist-to-hip circumference ratio decreased from 1.015 to 0.931, representing a decrease of 8.28%, and an obvious weight loss effect was achieved. After the intervention of the high-protein supplementation, the waist-to-hip circumference ratio in EGb decreased from 1.035 to 1.008, representing a decrease of 2.61%, and slight weight loss was achieved. WHR did not change significantly in CGc after three months (1.010 to 1.004). Numerous studies have demonstrated the substantial benefits of high-protein diets for obesity and metabolic health, as protein supplementation leads to a reduction in visceral fat mass and the activation of amino acid metabolism in the gut microbiota [59,60,61]. In addition, Zhang et al. demonstrated that soy protein supplementation can improve cardiovascular disease risk and significantly improve glucose metabolism, with potentially beneficial effects on diabetes [62]. Pal and Radavelli-Bagatini demonstrated that whey protein has a beneficial effect on reducing the symptoms of metabolic syndrome and cardiovascular risk factors [63]. According to the WHO’s recommendations, the standard value of WHR for males is 0.90, which shows that the WHR of the three groups was still greater than the standard value, indicating that they were still in the stage of obesity [58]. According to the WHO, standard BMI (kg/m^2^) is 18.5 to 24.9 for normal weight, ≥25.0 to 30.0 for overweight, and ≥30.0 for obese [59]. The EGa’s BMI dropped from 27.48 to 25.11, representing a decrease of 8.62%. The EGb’s BMI decreased from 27.53 to 27.09 kg/m^2^, representing a decrease of 1.60%. The BMI of the CGc decreased from 27.41 to 27.22, representing a decrease of 0.69 %. Overall, all the participants were still overweight, but their weight loss was consistent with results obtained from some academic studies. In the U.S., an obesity prevalence survey found a lower prevalence of obesity in people over 75 years of age compared with those 65–74 years old [60]. The gradual decline in body weight with age due to a reduction in lean body fat mass accounts for the lower incidence of obesity later in life [61,62]. Similarly, the study of Ford et al. confirmed that waist circumference increases with age, and WHR has been stated to be a better indicator of obesity, especially in older adults over 70 years of age [63]. Cartwright et al. suggested that starting in middle age, subcutaneous fat decreases and fat redistributes from the subcutaneous to visceral depots, leading to an increase in WHR [64].

Our study had some limitations. First, the majority of the study participants were healthy older adults. We had limited opportunities to recruit older adults in nursing centers, so recruitment was conducted in rural neighborhoods. Second, there are differences in the daily life patterns of older adults recruited in non-nursing centers from those of elders recruited in nursing centers, and this study could have used a cross-sectional design to limit any causal inferences. Finally, although all the participants signed an informed consent form, which included following a complete daily diet, the elderly participants attended family gatherings or went out for social events for small amounts of time. The fact that we could not entirely control diet was an important limitation of this study. Despite these limitations, this study included an experimental sample of older adults from rural Taiwan, where intervention with a physical activity program and high-protein supplementation was an objective measure, and the data collection ensured that information was obtained from all participants.

## 5. Conclusions

To sum up, this study shows that older adults in rural areas have insufficient knowledge about health care and do not understand the extended physiological symptoms of aging. Additionally, due to young and middle-aged males and females in rural areas joining the labor market, they spend less time caring for older adults at home. Therefore, the government must promote the establishment of long-term care centers or the establishment of activity centers and health consultation centers for older adults in rural areas to handle various physical activity plans and provide medical care knowledge, which will have a positive effect on the care of older adults from rural areas.

## Figures and Tables

**Table 1:** Basic participant information.

Variable	EGa (n = 20) M ± SD	EGb (n = 20) M ± SD	CGc (n = 20) M ± SD	*F* Value	*p*-Value
Age (years)	77.2 ± 5.3	78.5 ± 6.7	76.9 ± 6.1	0.94	0.273
Height (cm)	168.7 ± 8.2	169.2± 7.9	169.1± 7.4	1.13	0.102
Weight (kg)	78.2 ± 8.6	78.8 ± 7.8	78.4 ± 8.3	1.07	0.126

EGa means experimental group A, EGb means experimental group B, and CGc means control group C.

**Table 2:** Physical activity program.

Course	Posture and Motions	Operation Method
Cardiopulmonary function	Step-ups 20 (times) × 3 (set)	30 cm-high steps; go up the steps with one foot, stand with both feet together, go down the steps with one foot, stand with both feet together and repeat.
Lower body strength	Chair squats 15 (times) × 3 (set)	Place a chair behind you and start the standing position; squat down and raise your hands horizontally, and then squat down and touch the chair with your buttocks and immediately rise into a standing position. Do this once.
	Pistol squat 10 (times) × 3 (set)	Stand on one foot and leave the other foot off the ground (you can hold a support), and perform 10 deep squats on the left and right feet.
	Standing lunges 20 (times) × 3 (set)	Start in a standing position; put your feet together. Step on one foot (with a stride of more than 60 cm), retract the leg that has been stepped out, and step out with the other foot. Repeat the motion with your left and right feet 20 times.
	Walk in place with high legs 20 (times) × 3 (set)	Start a standing position, raise the leg in place up to thigh level, and repeat the operation with the left and right feet 20 times.
Upper body strength	10-pound dumbbell arm curls 12 (times) × 3 (set)	Stand vertically with dumbbells in your hands; and abduct your hands to a horizontal level at the same time.
	10-pound dumbbell flyers 12 (times) × 3 (set)	Stand vertically with dumbbells in your hands and abduct your hands to a horizontal level at the same time.
	10-pound dumbbell shoulder raises 12 (times) × 3 (set)	Hold the dumbbells at shoulder height as the starting point, raise both hands vertically at the same time, and then return to the starting point.

**Table 3:** Brief description of the test items.

Assessment Category	Test Item	Test Description
Body composition	BMI BFP BFM BMR SMM	Body composition was measured using an InBody 520 (Biospace Co., Ltd., Seoul, Korea). The InBody 520 estimates the composition of the human body based on bioelectrical impedance analysis (BIA). The bioelectrical resistance method is useful in body composition research because the electrode that is in contact with the human body measures the resistance value (impedance) of the body with an electrical current [36].
Body type	Waist and hip circumferences	Waist circumference (WC) and hip circumference (HC) were measured using constant tension measuring tapes with an accuracy of 1 mm (model: Orbitape). WC was measured at the midpoint between the last rib and the iliac crest. HC was measured from the maximum circumference behind the hip and anterior to the pubis [37]. Waist and hip circumferences were measured twice and averaged (cm).
Upper body strength	Handgrip strength	Handgrip strength (HS) was measured using a digital handheld dynamometer (Jamar ^®^) aligned with the forearm. Participants sat upright in an armless chair with their forearms parallel to the ground and elbows flexed at 90°. Participants were asked to squeeze the handle as hard as possible. Each limb was measured twice. Average of four measurements was taken (average of right and left hands).
	30 s dominant arm curl	Number of bicep curls in 30 s holding a hand weight (male 3 kg) [35].
Lower body strength	30 s sit to stand	Number of full stands in 30 s with arms folded across chest [35].
	2 min step	Number of full steps completed by raising each knee to point midway between the patella and iliac crest (number of times knee reaches target) in 2 min [35].

BMI means body mass index, BFP means body fat percentage, BFM means body fat mass, BMR means basal metabolic rate, and SMM means skeletal muscle mass.

**Table 4:** Descriptive statistics for the three experimental groups pre-test and post-test.

Variables	CGc (n = 20)		Imp. (%)	EGb (n = 20)		Imp. (%)	EGa (n = 20)		Imp. (%)
	Pre-Test	Post-Test		Pre-Test	Post-Test		Pre-Test	Post-Test	
BMI, kg/m^2^	27.41 ± 2.76	27.22 ± 2.53	−0.69	27.53 ± 2.36	27.09 ± 2.34	−1.60	27.48 ± 2.37	25.11 ± 2.24	−8.62
BMR, kcal	1512 ± 57.34	1503 ± 62.11	−0.60	1510 ± 59.54	1528 ± 61.71	1.19	1517 ± 59.53	1552 ± 65.62	2.31
BFP, %	27.77 ± 6.13	28.05 ± 4.24	1.01	28.01 ± 5.61	27.31 ± 5.97	−2.50	27.97 ± 6.08	25.37 ± 5.06	−9.30
SMM, kg	27.35 ± 2.67	27.07 ± 2.53	−1.02	26.84 ± 2.75	27.35 ± 2.46	1.90	27.41 ± 2.64	29.85 ± 2.13	8.90
BFM, kg	20.54± 1.87	20.21± 1.71	−1.61	20.62± 1.75	20.14 ± 1.58	−2.33	20.68± 1.65	18.77± 1.52	−9.24
WC, cm	114.6 ± 14.3	113.1 ± 15.1	−1.31	112.9 ± 12.5	107.1 ± 11.6	−5.14	112.3 ± 11.3	97.1 ± 10.7	−13.54
HC, cm	113.4 ± 11.7	112.7 ± 10.5	−0.62	109.1 ± 13.4	106.3 ± 12.2	−2.57	110.6 ± 11.7	104.3 ± 10.5	−5.70
HS, kg	15.16 ± 0.65	14.95 ± 0.72	−1.39	15.09 ± 0.84	16.42 ± 0.61	8.81	15.13 ± 0.82	18.59 ± 0.58	22.87
Arm curl, times	22.33 ± 2.95	23.26 ± 2.81	4.17	21.34 ± 2.56	25.15 ± 3.17	17.85	20.40 ± 2.84	28.25 ± 2.61	38.48
Sit to stand, times	18.43 ± 2.74	19.12 ± 2.85	3.74	18.68 ± 2.95	22.54 ± 2.95	20.66	18.55 ± 2.57	25.50 ± 2.73	37.47
2 min step, times	121.3 ± 7.45	120.8 ± 6.52	−0.41	122.6 ± 6.43	127.5 ± 7.67	4.00	120.3 ± 6.91	136.5 ± 6.54	13.47

Values are presented as means ± standard deviations. Imp: Improvement, EGa: experimental group A, EGb:experimental group B, CGc: control group C.

**Table 5:** Pre-test and post-test t-test results for all test items for the three study groups.

Variables	CGc-Pre	EGb-Pre	EGa-Pre
	CGc-Post	EGb-Post	EGa-Post
BMI, kg/m^2^	0.33 (0.54)	1.03 (0.11)	5.71 * (0.004)
BMR, kcal	0.27 (0.62)	1.69 (0.10)	5.25 * (0.008)
BFP, %	1.27 (0.19)	0.83 (0.24)	6.37 * (0.002)
SMM, kg	0.65 (0.38)	1.47 (0.12)	7.56 * (0.001)
BFM, kg	1.09 (0.26)	1.15 (0.14)	5.45 * (0.005)
WC, cm	0.47 (0.45)	0.58 (0.39)	4.39 * (0.009)
HC, cm	0.91 (0.29)	1.03 (0.17)	5.38 * (0.005)
HS, kg	0.66 (0.34)	1.89 (0.09)	4.57 * (0.007)
Arm curl, times	0.83 (0.32)	3.12 * (0.008)	8.82 * (0.0008)
Sit to stand, times	1.71 (0.09)	3.07 * (0.009)	13.75 * (0.0001)
2 min step, times	1.18 (0.13)	2.16 (0.09)	11.43 * (0.0006)

* *p* < 0.05. Values are presented as t-test statistics. EGa means experimental group A, EGb means experimental group B, and CGc means control group C. BMI means body mass index, BFP means body fat percentage, BFM means body fat mass, BMR means basal metabolic rate, SMM means skeletal muscle mass, WC means waist circumference, HC means hip circumference, and HS means handgrip strength.

**Table 6:** One-way ANOVA and LSD of post hoc test and post-test for test items.

Variables	CGc Post	EGb Post	EGa Post	*F (p)* Value	LSD
BMI, kg/m^2^	27.22 ± 2.53	27.09 ± 2.34	25.11 ± 2.24	3.88 * (0.007)	EGa > EGb = CGc
BMR, kcal	1516 ± 62.11	1528 ± 61.71	1542 ± 65.62	6.77 * (0.0008)	EGa > EGb = CGc
BFP, %	28.05 ± 4.24	27.31 ± 5.97	25.37 ± 5.06	5.48 * (0.003)	EGa > EGb = CGc
SMM, kg	27.07 ± 2.53	27.35 ± 2.46	29.85 ± 2.13	4.15 * (0.007)	EGa > EGb = CGc
BFM, kg	20.21± 1.71	20.14 ± 1.58	18.77± 1.52	3.68 * (0.01)	EGa > EGb = CGc
WC, cm	113.1 ± 15.1	107.1 ± 11.6	97.1 ± 10.7	6.03 * (0.001)	EGa > EGb = CGc
HC, cm	112.7 ± 10.5	106.3 ± 12.2	104.3 ± 10.5	5.44 * (0.004)	EGa > EGb = CGc
HS, kg	14.95 ± 0.72	16.42 ± 0.61	18.59 ± 0.58	3.85 * (0.009)	EGa > EGb = CGc
Arm curl, times	23.26 ± 2.81	25.15 ± 3.17	28.25 ± 2.61	5.26^*^ (0.002)	EGa > EGb > CGc
Sit to stand, times	19.12 ± 2.85	22.54 ± 2.95	25.50 ± 2.73	5.71 * (0.003)	EGa > EGb > CGc
2 min step, times	120.8 ± 6.52	127.5 ± 7.67	136.5 ± 6.54	13.75 * (0.0002)	EGa > EGb = CGc

* *p* < 0.05. Values are presented as means ± standard deviations. EGa means experimental group A, EGb means experimental group B, and CGc means control group C. BMI means body mass index, BFP means body fat percentage, BFM means body fat mass, BMR means basal metabolic rate, SMM means skeletal muscle mass, WC means waist circumference, HC means hip circumference, and HS means handgrip strength.

**Table 7:** The differences in WHR in the experimental results.

Variables	Group	Pre-Test	Post-Test	Imp. (%)	*t* (*p*) Value	*F* (*p*) Value
WHR	EGa	1.015 ± 0.12	0.931 ± 0.05	−8.28	3.714 * (0.01)	5.651 * (0.002)
	EGb	1.035 ± 0.09	1.008 ± 0.11	−2.61	1.046 (0.08)	
	CGc	1.010 ± 0.07	1.004 ± 0.06	−0.59	0.293 (0.41)	

* *p* < 0.05. WHR is presented as waist-to-hip ratio. Improvement is presented as Imp.

## Data Availability

The experimental results obtained real data, and study participants’ pre-test and post-test data were obtained after training. Participants agreed with the data structure via a confirmation form, and the confirmation included disclosure upon reasonable request. All the datasets on which the conclusions of the paper rely are available to editors, reviewers, and readers.
